# Correction: Membrane technologies in toilet urine treatment for toilet urine resource utilization: a review

**DOI:** 10.1039/d3ra90010j

**Published:** 2023-02-06

**Authors:** Chengzhi Yu, Wenjun Yin, Zhenjiang Yu, Jiabin Chen, Rui Huang, Xuefei Zhou

**Affiliations:** a State Key Laboratory of Pollution Control and Resource Reuse, College of Environmental Science and Engineering, Tongji University Shanghai 200092 China zhouxuefei@tongji.edu.cn +86-21-6598-2693; b The Third Clinical Medical College, Zhejiang Chinese Medical University Hangzhou 310053 China; c Shanghai Institute of Pollution Control and Ecological Security, Tongji University Shanghai 200092 China

## Abstract

Correction for ‘Membrane technologies in toilet urine treatment for toilet urine resource utilization: a review’ by Chengzhi Yu *et al.*, *RSC Adv.*, 2021, **11**, 35525–35535, https://doi.org/10.1039/D1RA05816A.

The authors regret that there are errors in the referencing in the original tables. The corrected versions of Tables 2, 3, 4 and 5 are shown below.

**Table 1:** The application of external pressure-driven membrane technologies^a^

Process	RC	Target	Performance	Reference
RO	HU	Urea and ammonia retention	64% unionized ammonia, 93% TOC retention	47
RO	FU		57% urea retention, ≥92% TOC retention, 86% conductivity decrease	47
RO	FU and HU, pH 9	P recovery	2.58 kg and 1.24 kg precipitates from 1 m^3^ HU and FU, precipitated solids contain 8.1–19.0% P, 10.3–15.2% Ca, 3.7–5.0% Mg and 0.1–3.5% ammonium nitrogen	50
RO	Mixed water	Water recovery	87 ± 5% water recovery	52
NF	HU, pH 11.5		90% unionized ammonia recovery, 98% TOC retention	47
NF	FU, pH 5	Urea retention	56% urea retention, ≥92% TOC retention, 96–97% conductivity decrease	47
RO-MBR	FU	N removal and P recovery	90% phosphorus recovery, 45% nitrogen removal	51

a RC – reaction conditions, FU – fresh urine, HU – hydrolyzed urine.

**Table 2:** The application of vapor pressure-driven membrane technologies^a^

Process	RC	Target	Performance	Reference
DCMD	FU	Volume reduction and nutrient concentration	Urine concentrated 17.8 times, 97% P and K rejection	18
DCMD		Specific ammonia transfer inhibition	SAT was reduced to 6.91 × 10^−5^ g-N per g-H_2_O	60
MD	HU, pH 10, water vapor gradient 30 °C	Water recovery	80% water recovery, 98% of TOC, 98% of Na^+^, 89% of K^+^ rejected	63
IMD-AC	HU	Ammonia recovery	60% ammonia recovery, 95% energy saving	64
FO-MD	FU	Water recovery	98% TOC, TN and NH_4_^+^ removal	34
FO-MD	FU and HU	Water recovery	Water flux of 31.5 (FU) to 28.7 (HU) L m^−2^ h^−1^	65
MD-MBR	HU	Non-odorous high-concentration liquid fertilizer production	Total dissolved solid concentration of 280 g L^−1^	66

a RC – reaction conditions, FU – fresh urine, HU – hydrolyzed urine.

**Table 3:** The application of chemical potential-driven membrane technologies^a^

Process	RC	Target	Performance	Reference
FO	HU	Volume reduction	The urine volumes were reduced to 1/2–1/5	19
FO	FU	Ammonia recovery	86% recovery of ammonia	75
	DS pH < 6.5			
	FS pH > 11			
FO	FS	N, P recovery	40% N recovery, 50% P recovery	76
FO	FS	Urine concentration	50% N recovery, 93% P recovery, economic benefits are 5.3 times the running cost	77
FO	HU	Water recovery	89% TN rejection with 75% water recovery using 5 M NaCl as the DS, 97% TN rejection with 50% water recovery using 5 M glucose as the DS	78
FO	Cave exploration	Urine volume reduction	86% TN rejection with 75% water recovery	79
FO	FU, HU	*Chlorella vulgaris* culture dewatering	Algal concentration was increased four-fold	80
FO-MD	FU	Urea recovery	45–68% urea concentration with 90% TOC rejection	82

a RC – reaction conditions, FU – fresh urine, HU – hydrolyzed urine.

**Table 4:** The application of electric field-driven membrane technologies^a^

Process	RC	Target	Performance	Reference
ED		Nitrogen recovery	95.6% nitrogen recovery	87
EDMBR	HU	Phosphate and sulfate recovery	65% phosphate recovery, 54.9% sulfate recovery	88
MBR-ED	FU	Urine treatment	80% ion collection	86
RED	FU and HU	Energy recovery	A maximum *E*_Net_ of 0.053–0.039 kW h m^−3^ of real urine	90
MD-RED		Water and energy recovery	47% Gibbs free energy recovery	91

a RC – reaction conditions, FU – fresh urine, HU – hydrolyzed urine.

The references themselves are correct and have not been adjusted from the original article.

The Royal Society of Chemistry apologises for these errors and any consequent inconvenience to authors and readers.

## Supplementary Material

